# Heart Surgery and Disruptive Technology

**DOI:** 10.21470/1678-9741-2019-0608

**Published:** 2019

**Authors:** Domingo M. Braile, Paulo Roberto B. Evora

**Affiliations:** 1 Editor-in-Chief - BJCVS Faculdade de Medicina de São José do Rio Preto (FAMERP), São José do Rio Preto, SP, Brazil and Universidade de Campinas (UNICAMP), Campinas, SP, Brazil.; 2 Editor-in-Chief Interim - BJCVS Faculdade de Medicina de Ribeirão Preto da Universidade de São Paulo (FMRP-USP), Ribeirão Preto, SP, Brazil.

Cardiac surgery has been considered the apogee of surgical procedures for over 40 years, occupying a noticeable place among the candidates for residency in the specialty, who were competing among the best brains of universities.

The characteristics of the surgical procedure required highly capable professionals, from a technical and tactical point of view, in a challenging environment in which patients’ lives were artificially maintained by equipment with highly complex technology that incorporated the most advanced knowledge about physiology and homeostasis of cell metabolism^[1]^.

The knowledge about physiology was tested in each intervention, forcing the professional to know deeply the functioning of the circulatory system and the organism entirely.

Each successful operation was a rewarding victory for the surgeon to such an extent that one of the most brilliant pioneers of the specialty, Prof. Denton Cooley, at a conference I attended, said that: Just the fact that the patient allowed himself to be operated was already a great reward for the surgeon.

History began to change in 1978, when the radiologist and cardiologist Andreas Grüntzig, or Andreas Gruentzig, assisted by an engineer, Heinrich Hopf, miniaturized a balloon catheter, which allowed him to dilate stenosed coronary arteries^[2-9]^.

This concept already existed, since Charles Dotter, along with Melvin Judkins, had published in 1964, in the journal Circulation, the dilation of peripheral arteries by the passage of coaxial catheters of progressive diameters, saving 11 patients with obstruction of lower limb arteries from gangrene.

It was created the concept of “transluminal angioplasty”^[7]^.

Surprisingly, due to complications at the beginning of the experiment, the procedure was abandoned and much criticized in the United States of America, where Dr. Dotter was nicknamed “crazy Charlie”^[7]^.

Nevertheless, the concept of dilating arteries remained.

Dotter himself and other German surgeons, such as Dr. Eberhart Zeitler, have considered using inflatable balloons to compress atherosclerotic plaques.

The balloons already existed, because an American, Dr. Thomas Fogarty, had created a catheter with a balloon to perform embolectomies^[7]^.

This was the status of the treatment of arterial obstruction, when a charismatic figure emerged, which would revolutionize the treatment of coronary artery obstruction.

It was a “disruptive technology”.

Myocardial revascularization, which represented about 50% of the cardiac surgeon’s work, started to compete with a less aggressive technique performed by a simple incision or even a puncture of a peripheral artery, without the need for anesthesia or transthoracic incision and extracorporeal circulation for saphenous vein grafts, mammary artery anastomoses, or other arterial grafts, to the coronary vessels, bypassing the obstructive lesions.

Grüntzig’s coronary angioplasty evolved into new variants with the introduction of “stents”, small metal structures introduced into the arteries by dilating them to avoid restenoses, which frequently occurred after some time of dilation^[7]^.

Stents kept being refined, and drug eluted to prevent cell proliferation that would cause stenosis.

In 1999, pioneering in Brazil, Dr. José Eduardo de Sousa^[8]^, at the Instituto Dante Pazzanese de Cardiologia, implanted the first stent coated with an antiproliferative drug, the sirolimus, and it was the first-in-man application worldwide.

These new classes of stents kept being improved, competing head-to-head with myocardial revascularization operations.

The technology continues to evolve, because complications persist, and myocardial revascularization is still the gold standard in more complex cases^[9,10]^.

Dilation of stenosed coronary arteries with balloons was followed by implantation of metallic stents to avoid frequent restenoses.

As the problem had not been solved, pharmacological stents, eluted with antiproliferative drugs, were developed to increase patency in medium and long terms.

These procedures constitute what is conventionally defined as “disruptive technology”.

“Disruptive technology is a term that describes technological innovation, with characteristics that cause a disruption of established patterns, models, or technologies”^[11]^.

That’s how myocardial revascularization operations were being replaced by a new procedure, without cardiovascular surgeons becoming aware that their work was being threatened by a less invasive technique, assimilated by other professionals, and preferred by patients^[7,8]^.

As quoted textually by Dunning^[11]^, in 2019, “…All these systems require general anesthesia and we must learn the lessons of the cardiac surgeons who were slow to enter the catheter labs and who lost the leadership in TAVI and revascularization.”.

To cite examples, I refer to the transcatheter valve implant, and its abbreviations “TAVI” (transcatheter aortic valve implantation) and “TAVR” (transcatheter aortic valve replacement)^[12-14]^.

The replacement of heart valves was absolutely established with the operations, extracorporeal circulation, and surgical replacement of stenosed, insufficient, or double-injured valves by metallic or biological prostheses.

However, elderly patients with aortic valve stenosis posed a high risk for enduring a conventional heart surgery in 2002.

In that year, Alain Cribier^[12]^, after studies on cadavers and animals, made the first catheter aortic valve implantation, in the city of Rouen, in a patient with severe calcified aortic stenosis, as a compassionate procedure, because of the critical condition of the patient

A crimped (crushed) biological prosthesis on a balloon was used to dilate the stenosed valve and at the same time to implant the valve inside the native valve, anchoring it over the calcium deposits.

Some other cases were performed on other compassionate patients (almost dying), with acceptable results.

Few people gave credit to this disruptive technique, not believing in its viability.

Dr. Alain Cribier himself came to me, at that time in the city of São Jose do Rio Preto, so that he could develop a properly treated bovine pericardium valve inside the support he had already developed.

At the time, I arranged a meeting between Dr. Cribier himself, the hemodynamicists Dr. Moacir Godoy and Dr. Luiz Antônio Gubolino, and me, as a cardiovascular surgeon.

Honestly, that technique did not impress us favorably.

To collaborate with the researcher at Braile Biomédica®, it was developed a valve suitable for the metallic support.

At the inventor’s request, an engineer spent some time in the company to make sure of the quality of the procedure.

That engineer started producing the valves herself in Canada. And I never heard from Dr. Cribier again.

In 2004, a multinational industry bought the rights of the incipient industry led by Dr. Alain Cribier.

Since then, hundreds of thousands of patients, currently even of medium or low risk, have been treated by the new technique improved over time^[13,14]^.

Another example, still under development, is the implantation of transcatheter valves for mitral and tricuspid positions, a challenging field^[15]^.

Besides that, there is another disruptive technology, widely developed, namely valve-in-valve implants, which show consistent results in repairing damaged biological valves, replacing reoperations with advantages.

Surgeons, as it is often the case with professionals who have preconceived ideas and are developing their activity in a comfort zone, did not believe in the new techniques, which developed vigorously, to the point of changing the concept of “heart surgeon”, there being need for the advent of a new professional that I decided to call “new cardiac surgeon”^[16]^.

This specialist should be able to maintain his/her classic activities, and also introduce in his/her portfolio the new less invasive technologies.

Many of the operations to correct birth defects, such as atrial septal defect (ASD), interventricular septal defect (VSD), pulmonary valve stenosis (PVE), closure of persistent ductus arteriosus, and other cardiac defects, are gradually being treated by little invasive techniques^[17]^.

The “new cardiovascular surgeon” should have a more extensive education than that provided by the established residency until a few years ago.

Kotsis et al.^[16]^, in 2013, in their article “Application of See One, Do One, Teach One Concept in Surgical Training”, state: “Residents must be encouraged and given the opportunities to learn and improve upon their surgical skills by mentors who continue to improve upon their skills as well. This can all be done in an environment that keeps patient safety at the forefront”.

Vaughn A. Starnes, president of the American Association for Thoracic Surgery (AATS), celebrating the significant date of the 100^th^ Annual Meeting, reaffirms the principles that guided its founding at the beginning of the last century^[18]^.

- Build a resource that studies the disease and improves outcome measures.
- Simplify data collection to improve the accuracy and quality of the data.
- Serve all areas of the specialty, as well as multidisciplinary areas.
- Give contributors access to their own data.
- Provide a collaborative space for surgeons around the world to engage.

I hope that cardiovascular surgeons and residents of cardiovascular surgery can assimilate the messages specified here, expanding their knowledge for treatment of heart disease patients, with all current and future techniques.
